# Effects of Geometry and Supporting Silicone Layers on the Performance of Conductive Composite High-Deflection Strain Gauges

**DOI:** 10.3390/jcs8110467

**Published:** 2024-11-11

**Authors:** Hailey E. Jones, Spencer A. Baker, Jadyn J. Christensen, Tyler Hutchinson, Heather A. Leany, Ulrike H. Mitchell, Anton E. Bowden, David T. Fullwood

**Affiliations:** 1 Department of Mechanical Engineering, Brigham Young University, 350 Engineering Building, Provo, UT 84602, USA; 2 Department of Exercise Science, Brigham Young University, 106 SFH, Provo, UT 84602, USA

**Keywords:** high-deflection strain gauges, piezoresistance, viscoelasticity, nanocomposites

## Abstract

Piezoresistive sensors composed of nickel nanostrands, nickel-coated carbon fibers, and silicone can be used to measure large physical deflections but exhibit viscoelastic properties and creep, leading to a complex and nonlinear electrical response that is difficult to interpret. This study considers the impact of modifying the geometry and architecture of the sensors on their mechanical and electrical performance. Varying the sensor thickness leads to potentially significant differences in conductive fiber alignment, while adding external layers of pure silicone provides elastic support for the sensors, potentially reducing their extreme viscoelastic nature. The impact of such modifications on both mechanical and electrical behavior was assessed by analyzing strain to failure, the magnitude of hysteresis with cycling, the repeatability of the electro-mechanical response, the strain level at which resistance begins to monotonically decrease, and the drift in electrical response with cycling. The results indicate that thicker single-layer sensors have less electrical drift. Sensors with a multilayered architecture exhibit several improvements in behavior, such as increasing the range of the monotonic region by approximately 52%. These improvements become more significant as the thickness of the pure silicone layers increases.

## 1. Introduction

Traditional strain gauges, which typically utilize delicate metal components, can only endure up to ~5% strain before failure [1–3]. This limitation makes them unsuitable for applications requiring a large amount of deflection, such as compliant mechanisms, soft robotics, tactile sensors, and smart wearable devices [4–7]. A promising approach involves integrating rubber-like polymers with conductive fillers (e.g., carbon nanotubes, graphene, or metal nanowires) [6,8–13].

The strain gauges investigated in this study are composed of nickel-coated carbon fibers (NCCFs) and nickel nanostrands (NiNs) embedded in a silicone matrix. The ferromagnetic qualities of these sensors make them susceptible to a confounding factor of magnetic fields. However, in typical applications, these sensors are unlikely to encounter magnetic fields strong enough to significantly influence particle position compared with the applied mechanical stresses. The greater difficulty of these sensors is that polymer-based sensors exhibit both elastic and viscoelastic properties. The nanoparticles induce localized stress concentrations that amplify the viscoelastic response and increase viscoplastic creep. As a result, the electrical and mechanical behaviors of these high-deflection sensors are more complex than those of conventional metal strain gauges. These sensors have a highly time-dependent response that requires knowledge of current and recent conditions for accurate interpretation [9,14]. This study aims to improve such behaviors. The main objective is to improve the repeatability and interpretability of the electrical response of the sensors by adjusting sensor thickness and adding multilayered architecture, meaning external layers of silicone.

The assumed electrical behavior of the sensors correlates the measured resistance with the experienced strain. Electricity flows through the sensor by following paths of adjacent nanoparticles, as studied in Clayton and Johnson [3,15]. When the gap between these nanoparticles is on the order of nanometers, quantum tunneling allows the current to flow. The resistance across these nano-scale gaps dominates the overall sensor resistance [16]. As the sensor stretches, the relative positions and gap sizes between the conductive particles change, altering the resistance. Given the low thickness to fiber length ratio of the current sensors, one approach to improving electrical response is to produce thicker sensors, allowing a more 3D fiber structure. Another less direct approach is to modify the extreme viscoelastic mechanical response of the sensors.

**Figure 1.**
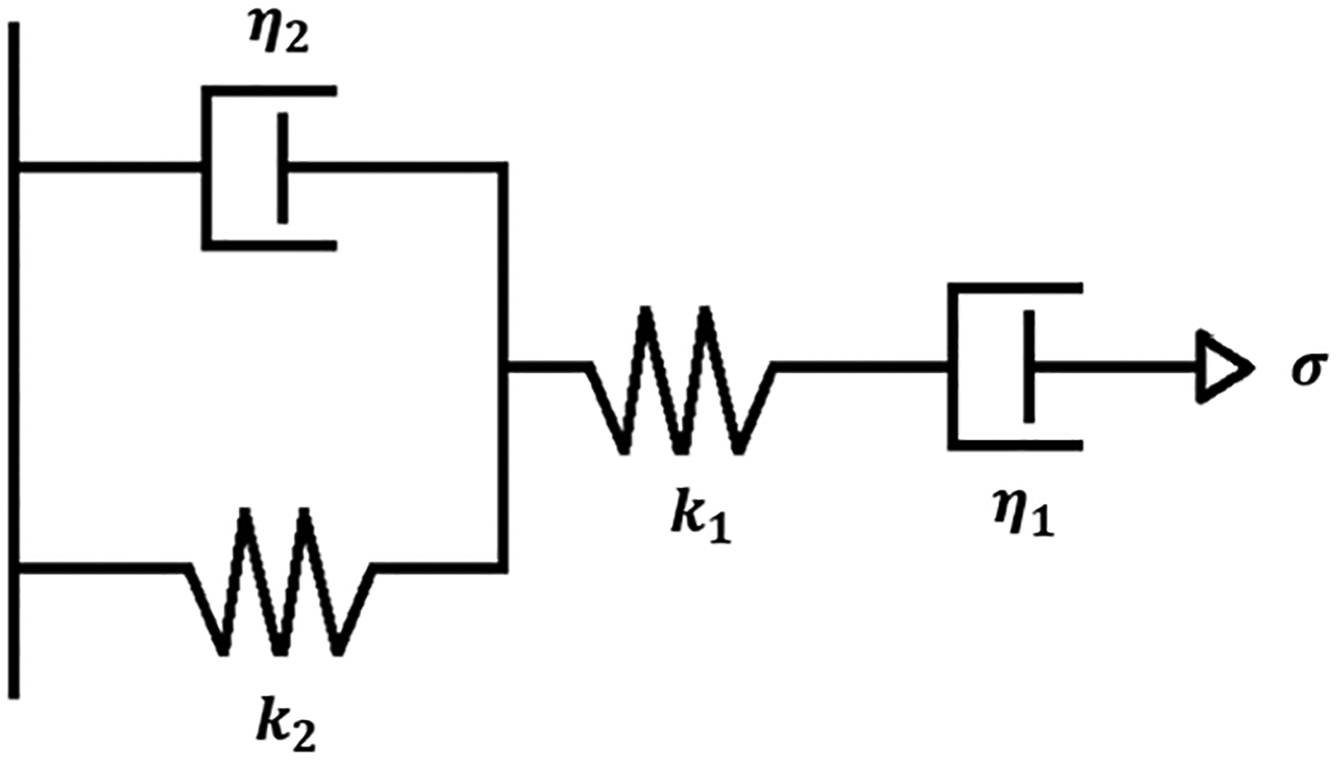
Burgers’ rheological model. *k*_1_ is the elastic spring constant, *η*_*1*_ is the viscoplastic damper coefficient, *k*_2_ is the viscoelastic spring constant, and *η*_*2*_ is the viscoelastic damper coefficient.

The mechanical behavior of a broad range of viscoelastic materials can be modeled using Burgers’ model [14,17–19]. In this model, depicted in Figure 1, springs and dampers are put in series to capture various properties: pure elastic behavior is a spring (spring constant, *k*_1_), viscoplastic behavior is a damper in series with this spring (damper coefficient *η*_1_), and viscoelastic behavior occurs as a spring (spring constant, *k*_2_) and damper (damper coefficient *η*_2_) in parallel.

The multilayered architecture seeks to curb the viscoelastic and viscoplastic response of current sensors. This approach adds layers of silicone on either side of the sensor. It is expected that the multilayer architecture would effectively add a spring in parallel to the entire Burgers model, ideally making the whole system more linearly elastic, like the silicone itself.

In summary, a more repeatable electro-mechanical response would enhance the sensors’ practical usability. This study investigates the influence of multilayer architecture and varied sensor thickness on sensor performance. Potential improvements are crucial for practical applications requiring high reliability and precision.

## 2. Materials and Methods

### 2.1. Manufacturing

The nanocomposite sensors studied here are composed of a blend consisting of 49% Ecoflex 00–30 silicone, 4.5% nickel-coated carbon fibers (NCCFs), 44.7% nickel nanostrands (NiNs), and 1.9% surfactant by weight. Ecoflex 00–30 silicone was purchased from SmoothOn Inc., Macungie, PA, USA, and the nanoparticles were purchased from Conductive Composites, Cleveland, UT, USA (parts 1AC1PC-1 AND 3AA150). The nanoparticles and surfactants were thoroughly mixed into the silicone matrix and then extruded into compression molds machined out of aluminum. Molds of different depths were used to produce sensors with thicknesses of 0.8 mm, 1.4 mm, 2.0 mm, 2.6 mm, and 3.2 mm.

**Figure 2.**
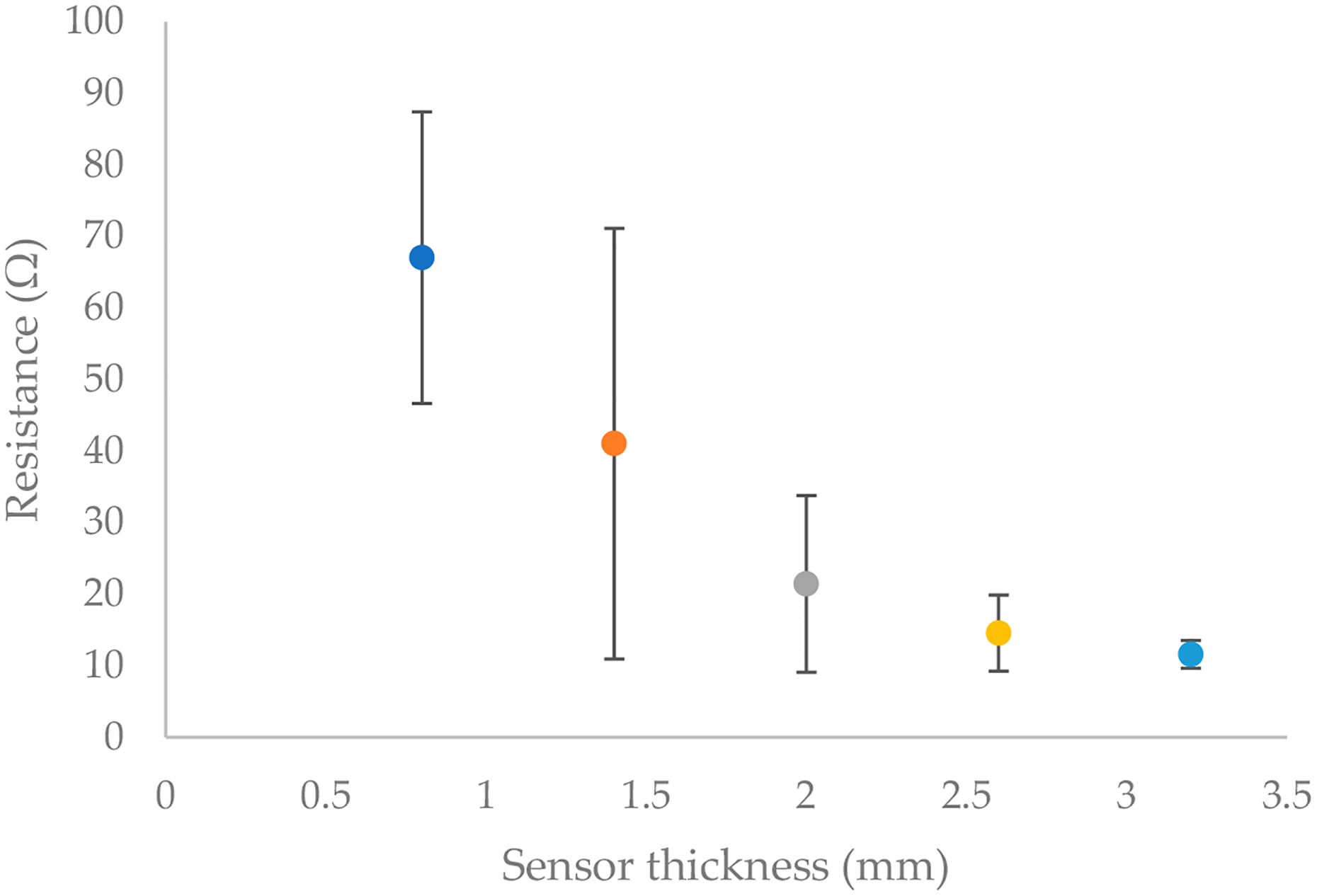
Average resistance at 20% strain for different sensor thicknesses of single-layer sensors.

To select a base sensor thickness for the multilayer architecture study, the electrical properties of the sensors had to be considered. As sensor thickness increases, the corresponding resistance decreases (see Figure 2). The range of resistance values offered by the 0.8 mm thickness sensors was within the range that the available electronics were already calibrated for. In addition, the higher resistance provided a wider range of resistances for the expected response, decreasing the potential impact of noise and equipment sensitivity. Therefore, when exploring a multilayer architecture, only 0.8 mm thickness for the initial single-layer sensor was utilized.

**Figure 3.**
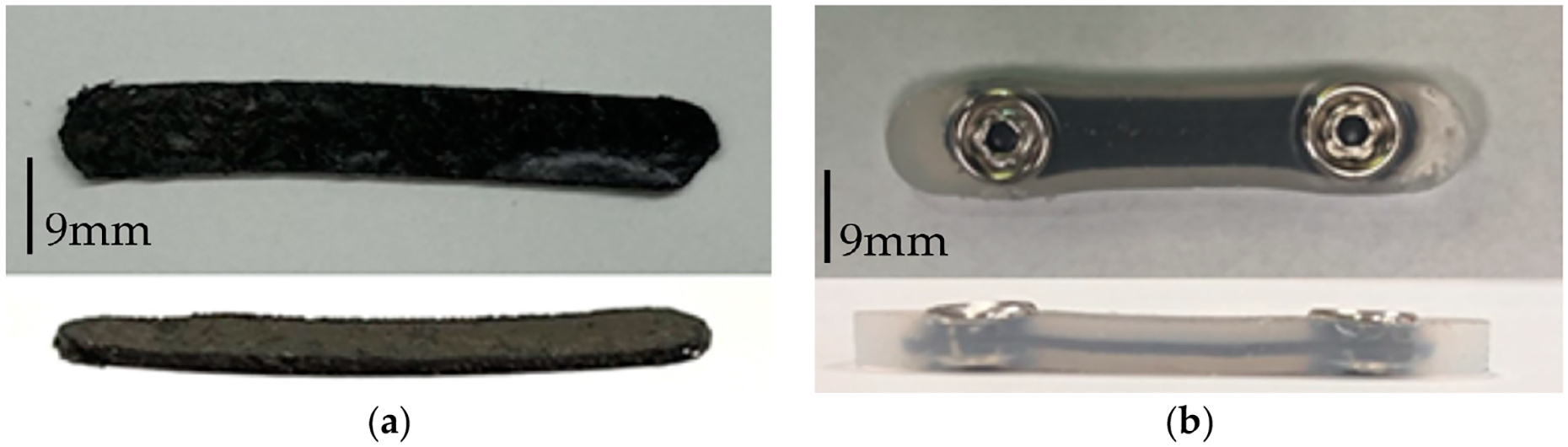
(**a**) Completed single-layer sensors. (**b**) Complete multilayer sensors with metal snaps.

The sensors (see Figure 3a) were initially cured at 88 °C under a vacuum pressure of 650 mmHg for 135 min. Following removal from the molds, they were then put through a secondary curing process at 192 °C for 30 min, except for the sensors which were to be used in the multilayer architecture, which were instead encased in Ecoflex silicone as described below.

The compression molds for multilayered sensors (see Figure 3b) varied in height: 1.35 mm for thin, 2.41 mm for medium, and 3.48 mm for thick sensors. The layering process involved filling the mold slightly less than halfway with silicone, which was allowed to cure at room temperature for 30 min. A thin layer of new silicone was subsequently added to allow better adhesion between the sensor and silicone layer. The sensor was centered on top of the new silicone and lightly pressed into place; this prevented bubbling from occurring. Following this, the remainder of the silicone was poured in. The sensor’s second cure of 192 °C for 30 min was then performed.

Due to the insulating properties of the silicone coating, 9 mm diameter metal snaps were installed on multilayered sensors to facilitate electrical connectivity. The prongs of the snaps penetrate the sensor, creating a pathway for conduction. They were installed symmetrically, 20 mm apart (dimensioned from the inner edges of the snaps). The snaps were purchased from TheSnapSource (Midland, MI, USA).

### 2.2. General Experimentation Approach

**Figure 4.**
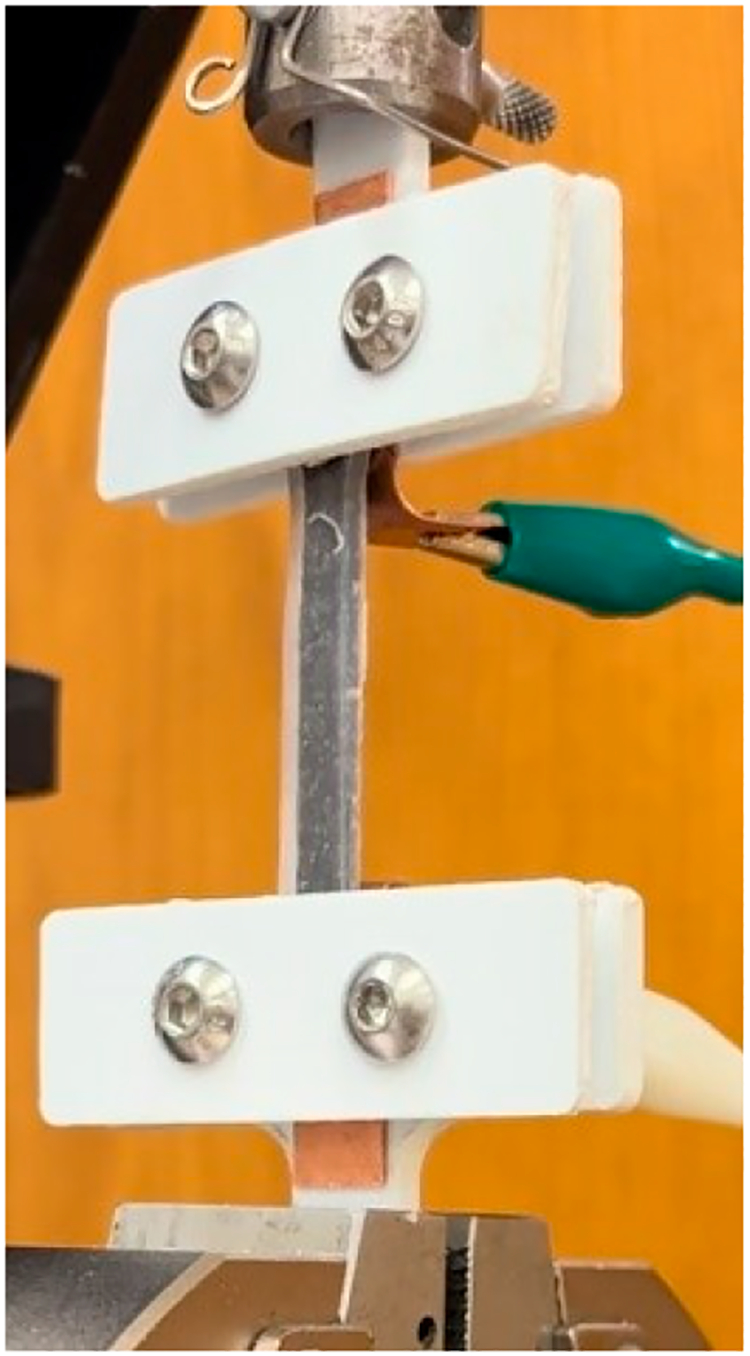
Multilayered sensor in Instron during testing.

In each test, an individual sensor was mounted into an Instron 3345 using polypropylene grips, as depicted in Figure 4. A sensor was placed vertically in the grips such that the unstressed length of exposed sensor was 20 mm. The sensors were centered on small copper strips glued to the polypropylene grips. These copper strips were bent at a right angle so that alligator wires could clip onto the copper strips. This provided an electrical pathway from the sensor to a Digilent Analog Discovery 2, which ran a 50 Hz square wave with an amplitude of 1 V, as programmed in WaveForms (v3.14.3), LabView was configured to read the output resistance using an NIH 9215 data-acquisition device.

The Instron measured the displacement and force applied to the sensor. The sensor was initially pulled to a 0.1 N pre-strain to eliminate slack, after which the force and displacement were zeroed, and LabView was set to record data. The specific test was then executed. All tests are described below.

### 2.3. Test Descriptions

#### 2.3.1. Strain to Failure

A sensor was pulled at a constant rate until failure at rates of 0.5, 0.05, or 0.005 mm/s.

#### 2.3.2. Mechanical Hysteresis Loop

For this test, a sensor was pulled at a constant rate until it reached 75% strain. Immediately, it was returned to 0% strain at the same rate. This cycle was performed five times for each sensor prior to the conclusion of the test. This was performed at rates of 0.5, 0.05, or 0.005 mm/s. The resulting stress-strain curves were plotted. For each cycle, the enclosed area of the stress-strain curve was normalized relative to that cycle’s maximum stress and compared across runs and sensors.

#### 2.3.3. Electro-Mechanical Cyclical Tests

Two significant factors that impact the usability of the sensors are the amount of electrical drift over repeated use and the range of strains that can be modeled in a one-toone function—this is also known as the monotonic region. To evaluate these characteristics, the electro-mechanical incremental strain test was designed to pull the sensor through a range of strains repeatedly. A sensor was pulled from 0% to 75% strain in 5% increments at a rate of 1 mm/s. At each increment, it was held at a constant strain for 3 s. After holding for 3 s at 75% strain, the same pattern was followed in reverse to return the sensor to 0% strain. This process was repeated 15 times for each sensor, with a 3 s pause between each cycle.

Displacement, force, and electrical resistance were extracted from the test data. The sensors exhibit an initial overshoot, or spike, in resistance corresponding to the initial change in strain [9]. The resistance decays exponentially to a steady-state asymptote which is approximated by maintaining strain for 3 s. The quasi-static resistance is recorded at the end of the hold. These quasi-static resistances were plotted relative to their strain and then connected with a spline for every cycle, creating resistance–strain curves as described by Baker [9].

Viscoelastic composites like our sensor often exhibit the Mullins effect [19], where the mechanical response during the first cycle is significantly different from subsequent cycles; hence, the first resistance–strain curve was assumed to exhibit atypical behavior and was ignored. The maximum resistance for the second and last cycles was determined. The difference between these resistance peaks was normalized relative to the final cycle’s maximum resistance to measure the drift in the sensors. This quantified the average percent change after 14 cycles.

#### 2.3.4. Electro-Mechanical Incremental Strain Tests

Like the electro-mechanical cyclic strain test, the electro-mechanical incremental strain test seeks to capture the electrical drift and monotonic region of a sensor after continual use; however, this test varies the maximum strain in each cycle to characterize the sensor’s dependency on time and previous loading. We began the test by pulling a sensor to a set strain and then relaxing it to zero. After 3 s of rest, the sensor was pulled to the set strain in 5% strain increments, holding for 3 s at every increment (including the set strain). The sensor was returned to a strain of zero in 5% strain increments, holding for 3 s at each increment. Lastly, the sensor was once again pulled to the set strain and then relaxed to zero. This entire procedure was performed at a rate of 1 mm/s and carried out seven times. A ‘cycle’ references one of the seven repetitions. The set strain was progressively increased from 15% to 75% in 10% increments for each cycle. Like the electro-mechanical cyclical test strain, the displacement, force, and resistance relative to time were extracted from the test data. The quasi-static resistance at each increasing increment was plotted with a spline for every cycle, creating a resistance–strain plot as described in [16]. These resistance–strain plots show the sensor’s dependency on past strains.

#### 2.3.5. Strain Prediction

The next step in sensor usability is to accurately interpret strain based on the output resistance. This interpretation is more likely to be accurate if sensors are created to have more consistent electro-mechanical behavior. To determine if multilayered architecture improved sensor consistency and interpretation, a model was created. This was achieved by using all the electro-mechanical cyclical tests for a given kind of sensor and normalizing each spline relative to its own critical resistance value. The splines were then evaluated at discrete resistance points between 0 and 1 and each point was averaged across all splines. In this average, any outliers were disregarded in the calculation. A new spline was made from the averaged points and used as the model for a given kind of sensor.

**Figure 5.**
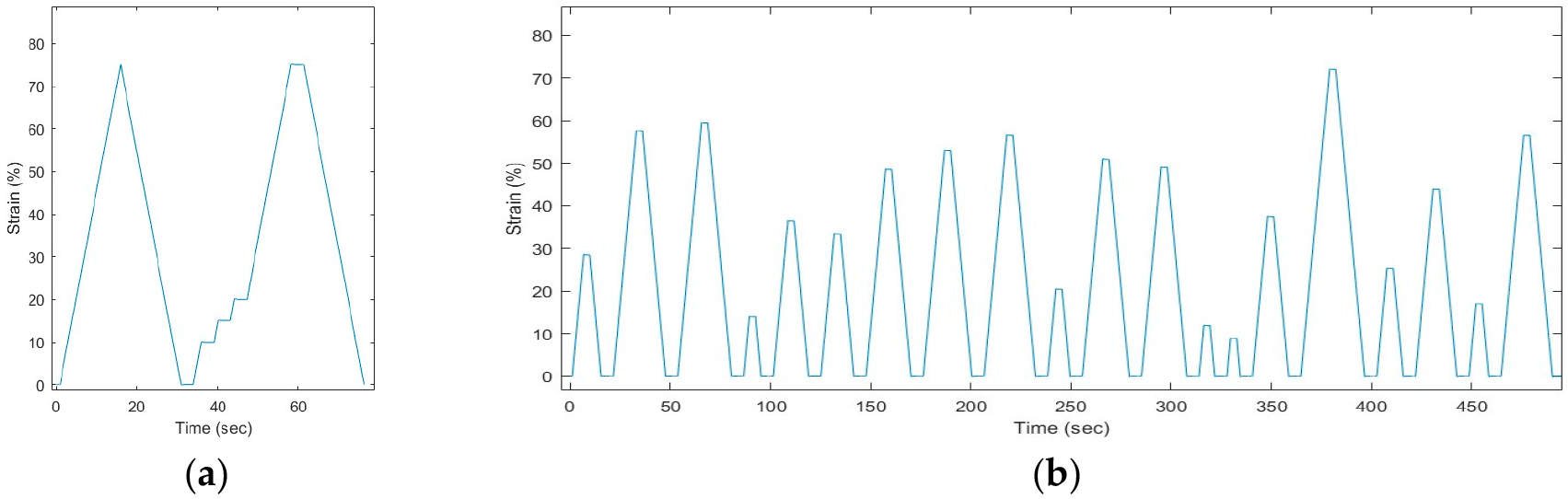
(**a**) The movement of the Instron for a multilayered calibration. (**b**) The movement of the Instron to 20 random strains for one sensor.

New sensors were then tested in the Instron. An example of the strain over time for a single sensor is shown in Figure 5. In this test, all rates were at 1 mm/s. The test began with the sensor being pulled to 75% strain and then immediately back down to zero. After 3 s of rest, the sensor was pulled to three distinct strains with a three-second pause between each strain. For single-layered sensors, this was at 20%, 25%, and 30% strain. For multilayered sensors, this was at 10%, 15%, and 20% strain. These strains are expected to contain the critical point, or the beginning of the monotonic region and maximum resistance that a sensor exhibits, due to the behavior seen in the electro-mechanical cyclical test.

After undergoing calibration, the sensors were stretched to 20 randomly determined strains. They were pulled at a constant rate, left at the strain for 3 s, then brought to 0% strain for another 3 s. This pattern was used for all 20 strains consecutively. An example of this is shown in Figure 5b.

After the test resistances were normalized, they were put into the model to estimate strain. These estimates were compared to the known strains and the R^2^ accuracy was calculated.

## 3. Results

### 3.1. Strain to Failure

**Figure 6.**
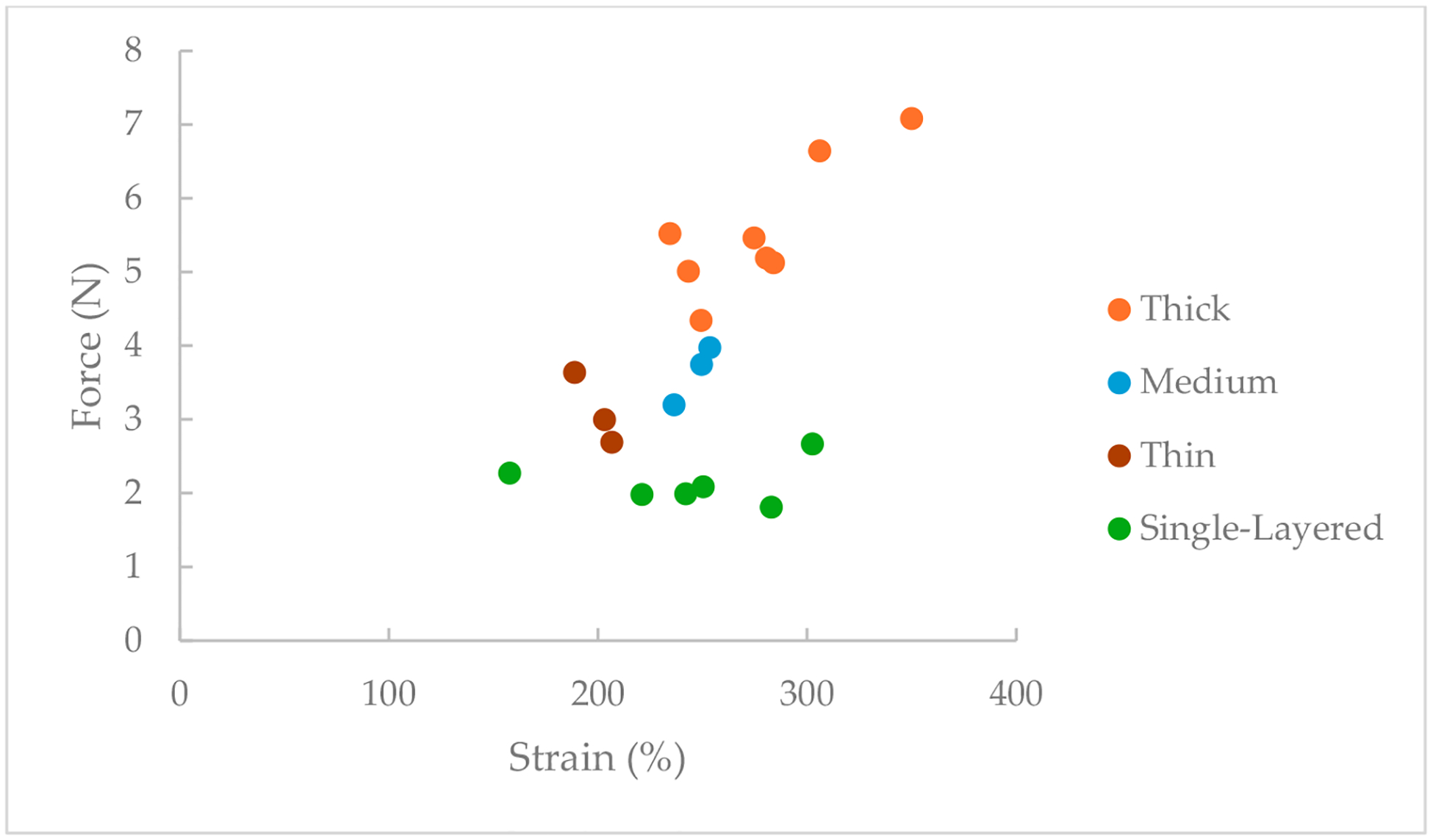
Point of failure for single- and multilayered sensors at all rates of pulling.

The strain to failure test compares the critical mechanical properties of single- and multilayered sensors. The test was carried out at four different rates; however, because there was no statistical significance in the correlation between the rate of pull and strain at failure, all rates are shown in Figure 6.

The statistics for the data in Figure 6 are recorded in Table 1. Because single-layered and thick multilayered sensors have large standard deviations relative to the mean values, it is difficult to prove statistically significant trends in failure strain. Potential causes for these variances include the material variance within the sensors themselves; as they are highcontrast composites, it is possible for small perturbations in the mixture structure to result in strain concentrations and subsequent failure. In addition, if the polypropylene grips exerted varied pressure on the sensors due to how much they were tightened, it could have caused deformation that weakened the sensor—this is particularly true for single-layered sensors which lacked the protective silicone barrier. Regardless, a comparison of the mean strain to failures across various sensors suggests that thicker external layers of silicone increase the likelihood of withstanding higher strains. It can be said with confidence that multilayered architecture preserves the high strain capabilities of single-layered sensors These sensors easily surpass the definition of a high-yield sensor, which is capable of ε > 100% [2].

Though the ability to withstand ε > 100% is preserved, to produce the same strain, the force required increases with the amount of silicone in the architecture. The difference between single-layered architecture (which required the least amount of force) and thick multilayered architecture (which required the most force) is about 3.63 N at failure on average (see Table 1). The increased stiffness in the sensor could skew the results in some studies by constraining the monitored object. For example, in a study like [17] where the sensors are used on the skin, the sensor may cause a significant increase in stiffness in the system, thus skewing the strain results. Though the force increases, the modulus of the added silicone is less than that of the nanoparticle layer. If the total cross-sectional area were to stay the same by decreasing the thickness of the nanoparticle layer when increasing the amount of silicone, it could mitigate the required force while increasing the elastic properties of the sensor.

### 3.2. Mechanical Hysteresis Loop

**Figure 7.**
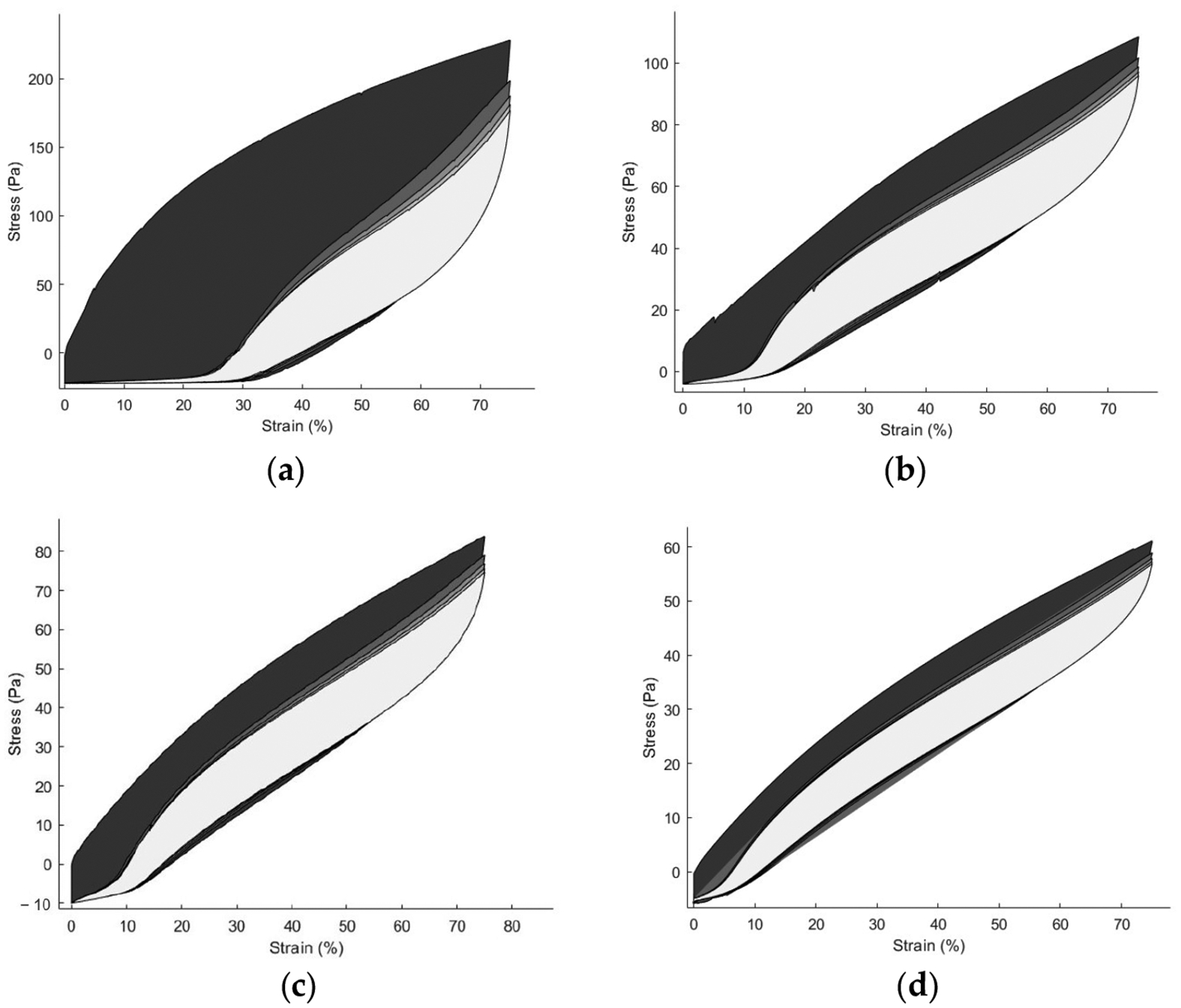
Stress–strain response of a sensor over cycling for a (**a**) single-layered, **(b**) thin multilayered, (**c**) medium multilayered, and (**d**) thick multilayered sensor. Darkest to lightest is the first to last cycle.

As discussed earlier, viscoelastic materials respond differently depending on their recent conditions. This can make the interpretation of sensor data more difficult. To compare variance in mechanical response, this test quantified viscoelasticity via the hysteresis loop area (see Figure 7). The hysteresis loop area describes the energy lost by the sensor during a cycle. It is captured by the stress–strain response of the sensor during cycling. Values are normalized relative to the cycle’s maximum stress and compared across samples. A larger change in normalized area from cycle to cycle indicates increased viscoelastic and viscoplastic deformation.

**Figure 8.**
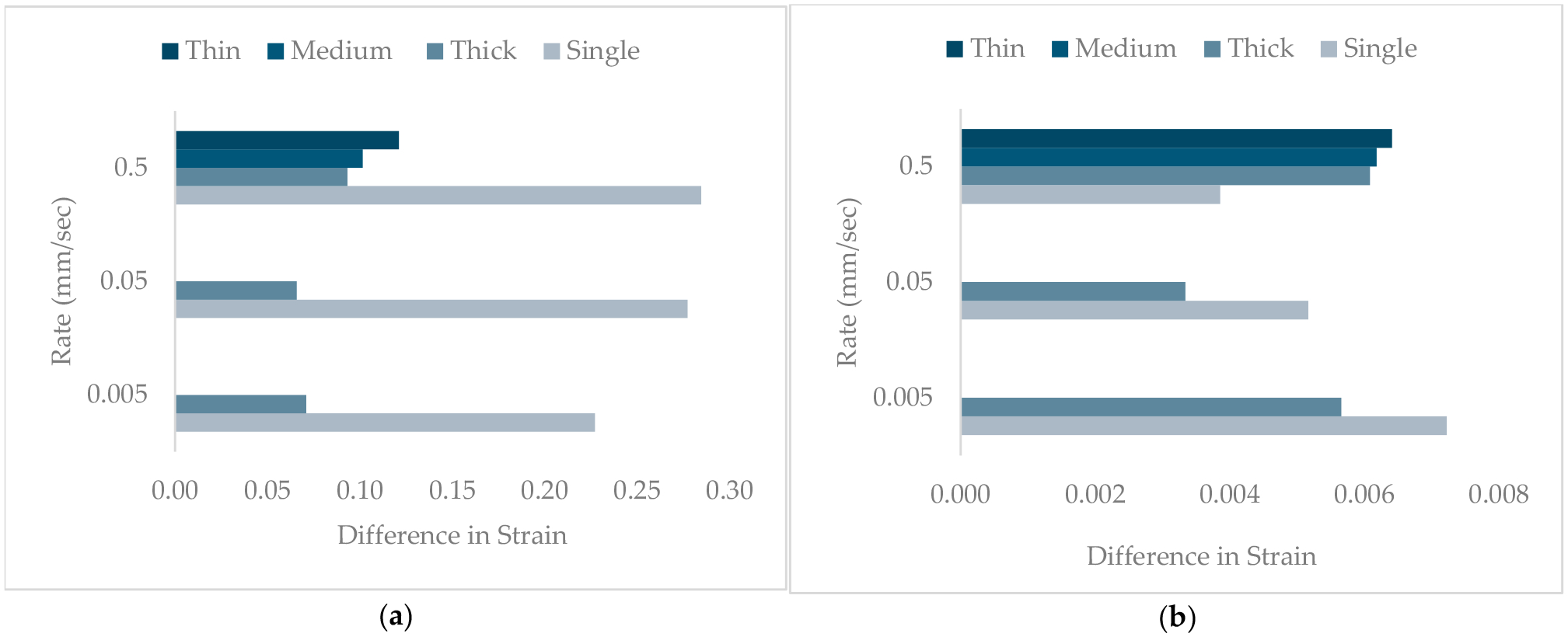
(**a**) Average difference in normalized area between the first and second cycle. (**b**) Average difference in normalized area from cycle to cycle. This was determined for cycles two through five.

The most significant change in area for all sensors occurs between the first and second cycle, as seen in Figures 7 and 8. This is evidence of the Mullins effect [20], which describes how viscoelastic materials exhibit a significantly different mechanical response during the first cycle compared to subsequent cycles. This variance can cause the sensor’s initial readings to be unreliable. Thus, mitigating the variance increases the sensor’s usefulness. Multilayered architecture successfully reduces the impact of the Mullins effect by approximately 74% when comparing thick multilayer with single-layer sensors. In addition, multilayered sensors have smaller hysteresis loop areas than single-layered sensors for the same cycle—this implies that the multilayered sensor is more likely to return to its original shape because it has a more dominant elastic response. However, as shown in Figure 8b, multilayered architecture (of any thickness) does not significantly decrease the average change in area from cycle to cycle. This means that multilayered sensors experience similar viscoplastic behavior and face challenges related to mechanical drift over time, just like single-layered sensors. In summary, while the multilayered architecture increased the elastic response and decreased the impact of the Mullins effect, it did not create statistically significant changes with regard to viscoplasticity and the resulting mechanical drift.

### 3.3. Electro-Mechanical Cyclical Tests

**Figure 9.**
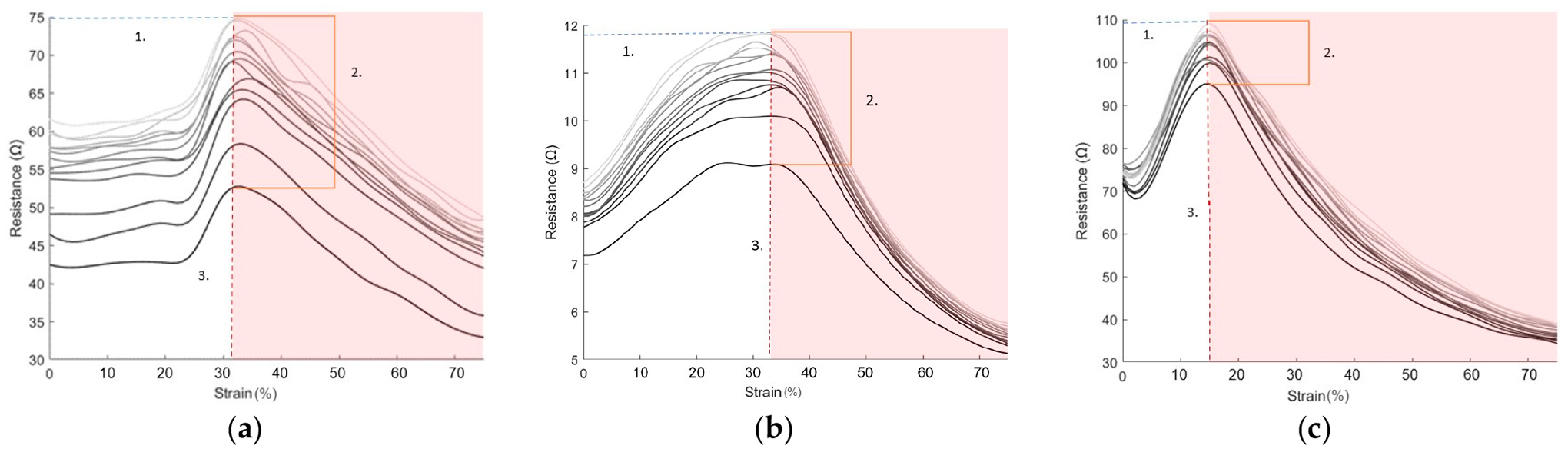
Resistance–strain curves for (**a**) single-layered, (**b**) 3.2 mm single-layered, and (**c**) thick multilayered sensor from cycle 2 to cycle 15. The fifteenth cycle is the lightest line, and the second cycle is the darkest line. Numerals on the images indicate 1. Maximum resistance 2. Drift magnitude 3. Strain at the critical point.

Resistance–strain curves characterize the relationship between applied strain and measured resistance for a sensor. Ideally, such curves would be time-independent, linear, and monotonic—this is not the case in reality. To create resistance–strain curves, quasi-static resistances are found at known strains. Since any rapid change in strain is accompanied by a short-lived positive spike in resistance, the resistance value is measured several seconds after the strain change to be closer to the true quasi-static resistance value. There are dynamic models capable of approximating the quasi-static resistance for less-controlled environments [9], but the quasi-static method was used in this study for simplicity and accuracy’s sake. The resistance–strain curve is drawn by connecting measured quasi-static points with a spline. In order to determine drift in resistance over repeated use, a curve is drawn for each individual cycle, as described in the Methods section. The first cycle is excluded due to the Mullins effect [20], as shown in the mechanical hysteresis loop test above. A few examples of the resistance–strain curves are displayed in Figure 9. The most notable features of these curves are the monotonic region, the critical point for each cycle, and electrical drift, as described below.

The monotonic region is the portion of the curve that can be approximated by a single-valued function (e.g., Burgers’ Model). It is highlighted by the pink areas in Figure 9. Any strains before the monotonic region cannot be captured by an invertible model, and therefore cannot be interpreted in practical use of the sensor. The critical point lies at the beginning of the monotonic region and coincides with the maximum resistance that a sensor exhibits. Ideally, the critical point would be at 0% strain so that the relationship between resistance and strain could be captured by a monotonic function for all strains. As can be seen, the strain value at which the critical value occurs remains constant across the cycles for all types of strain gauges.

Electrical drift describes the change in resistance at the critical point after repeated strain is applied. It is calculated by comparing the resistance of the critical point for the second cycle with that of the last cycle. It is normalized relative to the final resistance for comparative purposes. Decreased drift makes reliable modeling easier and therefore increases sensor practicality.

Table 2 indicates that for different thicknesses of single-layer sensors, the electrical drift generally decreases as the thickness increases. A notable improvement of 60% less drift occurs between the largest and smallest thickness. On the other hand, the critical strain appeared to increase marginally with single-layer thickness (7.2% overall).

When comparing the electro-mechanical behavior between multilayered architecture and single-layered architecture, significant improvements can be seen for the monotonic region. Given that the tested strains were capped at 75%, the range of strain captured by the monotonic region increased by 52% between the thick multilayered and single-layered sensor (see Table 3). However, the relative electrical drift was slightly worse by about 7.7%. This is contrary to the trend shown when comparing thick multilayered to thin or medium multilayered sensors, for which increased silicone correlates with improved behavior. This discrepancy could be due to variance in manufacturing (such as batch discrepancies or curing time) but deserves further study to draw firm conclusions.

### 3.4. Electro-Mechanical Incremental Strain Tests

**Figure 10.**
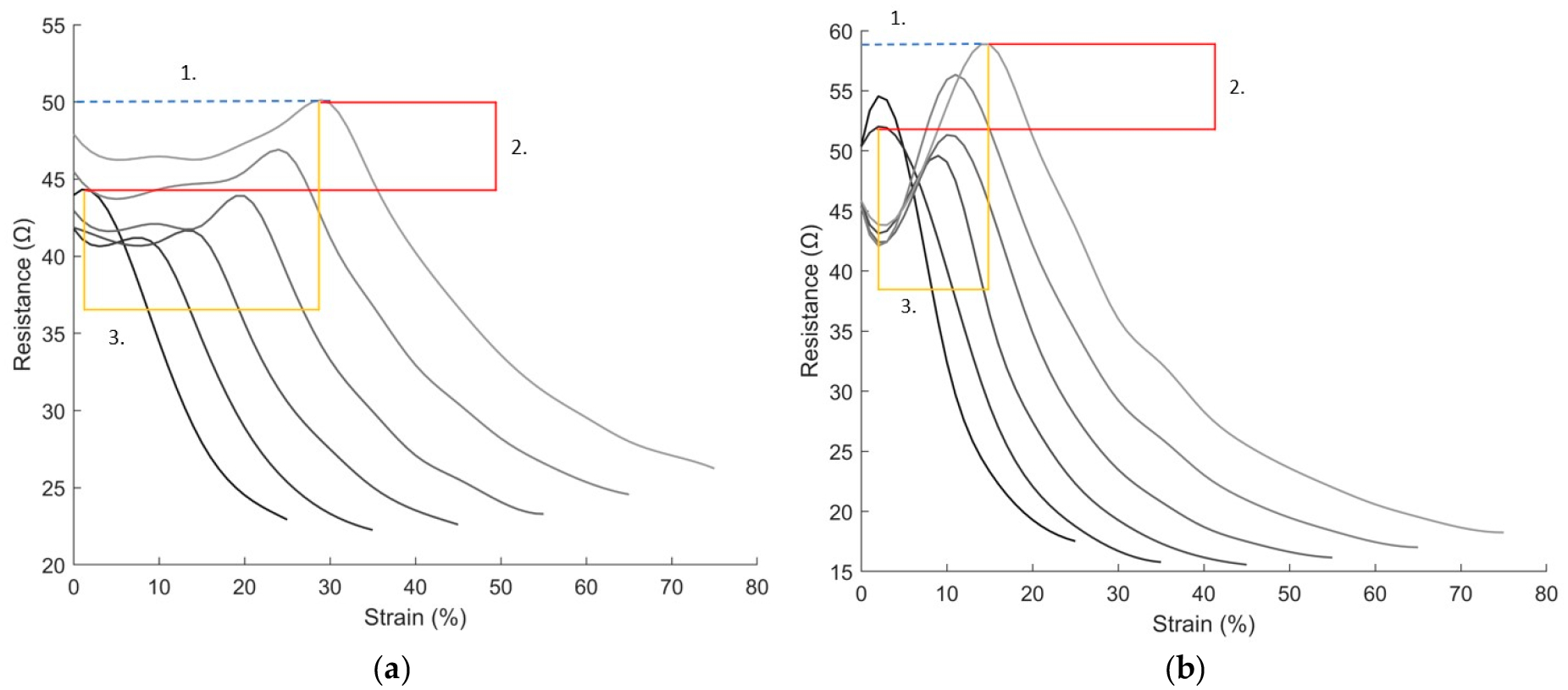
Resistance–strain curves. The second cycle is the darkest line, and the last cycle is the lightest line. Comparing 1. maximum resistance, 2. relative resistance drift magnitude, and 3. strain shift for (**a**) single-layer and (**b**) multilayer sensors, respectively. Average strain, force, and relative standard deviations at failure, disregarding rate.

Similarly to the electro-mechanical cyclical test, the electro-mechanical incremental test uses quasi-static values to create resistance strain curves. However, because of the change in the strains applied, as described in the Methods section, the resistance strain curves exhibited a drift in strain of the critical point (Figure 10). This drift shows the dependence of the sensor on previous conditions; as the maximum applied strain increases, the monotonic region decreases. When compared with Figure 9, it is clear that drift in the location of the critical point makes modeling the response more difficult. This is largely because of the dynamic monotonic region. If the history of a sensor becomes a required variable in the model, the usability of the sensor decreases; the model would become mathematically more complex, requiring increased computation time, and would be more vulnerable to error if historical data are missing. Multilayer architecture helps mitigate this issue. On average, multilayered sensors have 74% less drift in the position of the critical point (Table 4). However, the monotonic drift is not eliminated.

### 3.5. Strain Prediction

As described in the Methods section, the model used to predict strains averaged the resistance values of the electro-mechanical cyclical test data at distinct strain values for cycles 2–15 for all tests of a given sensor type. From these points, a spline was created; this spline was used to predict the strain based on the electrical resistance. One model was made for the single-layered sensors and one for the multilayered sensors.

**Figure 11.**
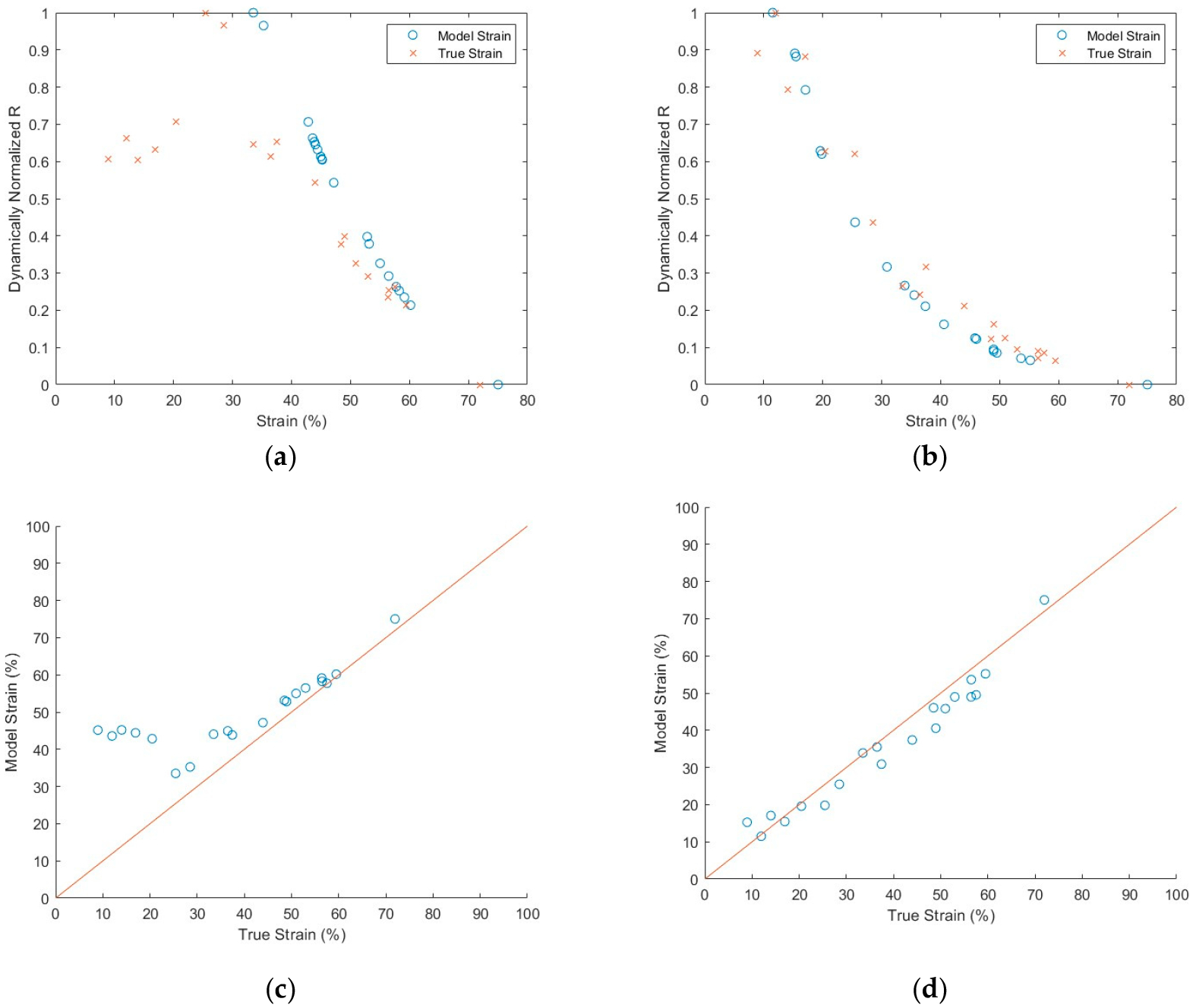
Comparing true and predicted strain. Graphs (**a**,**b**) compare known and estimated strain with normalized resistance for single- and multilayered sensors, respectively. Graphs (**c**,**d**) show an R^2^ representation, comparing true and modeled strain for single- and multilayered sensors, respectively.

A new batch of single- and multilayered sensors, which will be called validation sensors for clarity, were used to acquire data to test the model and determine the impact of all previous sensor behaviors. It is expected that the multilayered sensors are more likely to have accurate model predictions because of the decreased Mullins effect (see the results for the mechanical hysteresis loop) and the decreased electrical drift (see the electro-mechanical cyclical test). These improvements promote overall consistency in the electro-mechanical response. The validation sensors were stretched as described in the Methods section, with both the strain and resistance recorded. The normalized resistances were fed into the model to create an estimate of sensor strain at any given point. Figure 11a,b show the estimated and actual strains relative to the resistance of a validation sensor. Figure 11c,d directly compare estimated and actual strains for a validation sensor.

The accuracy of the estimated and true strains was greater for multilayered sensors than single-layered sensors, with an R^2^ value of 0.842 and 0.210, respectively. This is largely due to the increased monotonic region of multilayered sensors, a characteristic proven in the electro-mechanical cyclical test. The single-layered sensor becomes completely inaccurate under about 25% strain; because the multilayered sensor has a lower strain associated with the critical point, it is accurate for more of the tested strains. However, it is equally unreliable outside the monotonic region. This is why increasing the monotonic region to capture all possible strains is crucial. For single-layered sensors above 25% strain, the output becomes much more predictable, with an R^2^ value of 0.889. Thus, the phenomenological model used has similar accuracy for single- or multilayered sensors when looking at the monotonic region exclusively.

## 4. Discussion

Several tests were conducted to compare the mechanical and electrical properties of high-yield, nanocomposite sensors with varied architecture and sensor thickness to determine optimal performance. The results indicated that for the measured properties, increased sensor thickness generally improved sensor behavior. The most significant improvement is the 60% decrease in electrical drift between the largest and smallest single-layer thickness. While a slight increase was observed in the critical strain, the inclusion of multilayer architecture could counteract this effect.

When comparing various thicknesses of external silicon in the multilayered architecture, all tests suggested that thick sensors have the best behavior: they can withstand the greatest strain before failure, increased elastic response, have the largest monotonic region, and exhibit the smallest drift in resistance after cycling.

While thick multilayered sensors were the best performers, medium multilayered sensors had comparable results while requiring less material to produce. Medium sensors use about 36.6% less silicone than thick sensors, with the primary tradeoff being a 17.6% increase in electrical drift over 14 cycles. Thus, the architecture chosen should be specific to the application of the sensor. For example, a low-production test would likely use the thick architecture to ensure better sensor behavior. For tests with force-sensitive equipment or strict cost requirements, it may be that medium sensors are an excellent compromise between material cost and optimal sensor performance.

When comparing thick multilayered sensors with single-layered sensors, an analysis of the differences between key properties shows several significant improvements resulting from multilayer architecture. Key properties and their effects are laid out in Table 5.

Overall, the mechanical and electrical properties of the sensors were more repeatable when multilayered architecture was used. The cyclic viscoplastic drift decreased by 9.5% and the strain at failure was 14% greater. The electrical drift was somewhat inconsistent between various tests, with a 54% drift decrease in the electro-mechanical incremental test and a 7.7% drift increase in the electro-mechanical cyclical tests. This discrepancy is believed to be caused by the use of multiple batches in the electro-mechanical cyclical tests, which usually causes some variability in maximum resistance. Further testing is needed for a decisive conclusion on this factor. Regardless, the multilayered sensors captured a greater range of strains in every test. This can be seen in the strain prediction test, where the monotonic regions begin around 12% rather than 32% strain.

There are a few considerations related to the multilayered architecture; due to the increased amount of material, the sensor resists motion and requires up to 3× the force to move compared to single-layered sensors. This could become an issue when trying to measure physically delicate systems. This could be mitigated by decreasing the width of the sensor itself, thereby decreasing the overall material and elastic modulus. However, as was seen in the electro-mechanical cyclical test, decreasing the single-layer sensor thickness increases drift, which can become problematic.

## 5. Conclusions

Sensors capable of withstanding high deformation are necessary to allow for the measurement of systems with high deflection, such as soft robots, compliant mechanisms, and biomechanics. The nanocomposite sensors used in this study have proven to be a viable option to address this need, but still suffer from highly nonlinear, viscoelastic properties. While there are models that account for these behaviors, the accuracy can be increased by mitigating properties such as hysteresis and drift. This study proved that using multilayered architecture (meaning an encapsulating layer of silicone on the sensor) made the sensor more physically durable, electrically consistent, and capable of capturing small strains. It was noted that increasing sensor thickness decreased electrical drift, though it also decreased the monotonic region. The combination of architectural changes is dependent on the scenario in which the sensors will be used. In general, the thickest sensors and thickest multilayered architecture are recommended. However, both changes increase the overall thickness and stiffness of the sensor. Thus, if the application is used on a delicate system or if the application requires the sensors to have the smallest profile possible, then architectural modifications require further consideration. Overall, the changes proposed to the sensor architecture are expected to increase the accuracy of the measurement of high-yield systems.

## Figures and Tables

**Table 1.** Average strain, force, and relative standard deviations at failure disregarding rate.

Rate	Single-Layered	Thin Multilayered	Medium Multilayered	Thick Multilayered
Average Strain to Failure	2.42	1.99	2.46	2.77
Strain Standard Deviation	0.508	0.094	0.089	0.377
Average Force	2.13	3.10	3.63	5.54
Force Standard Deviation	0.302	0.482	0.399	0.89

**Table 2.** Average drift in resistance between cycle 2 and cycle 15 relative to maximum resistance. Average critical strain corresponds to the critical point.

Thickness	Average Drift	Average Critical Strain
0.8 mm	0.261 ± 0.11	0.345 ± 0.02
1.4 mm	0.191 ± 0.19	0.353 ± 0.03
2.0 mm	0.145 ± 0.09	0.356 ± 0.04
2.6 mm	0.177 ± 0.08	0.369 ± 0.07
3.2 mm	0.104 ± 0.08	0.370 ± 0.04

**Table 3.** Average drift in resistance between cycle 2 and cycle 15 relative to maximum resistance. Average critical strain corresponds to the critical point.

Sensor	Average Drift	Average Critical Strain
Single-layer	0.261 ± 0.11	0.345 ± 0.02
Multilayer Thin	0.413 ± 0.18	0.243 ± 0.10
Multilayer Medium	0.341 ± 0.11	0.213 ± 0.09
Multilayer Thick	0.283 ± 0.12	0.135 ± 0.05

**Table 4.** Average strain, force, and relative standard deviations at failure disregarding of rate.

Sensor	Average Resistance Drift	Average Critical Strain Drift
Single-layer	0.220	0.19
Multilayer	0.187	0.05

**Table 5.** Influences of multilayer architecture on several key sensor properties. Comparisons are made between single-layered and thick multilayered sensors. The change in properties are summarized by describing the level of impact (greatly decreased, decreased, no change, increased, or greatly increased) and whether or not that impact was beneficial (Yes or No).

Property	Multilayer Effect on Property	Improvement?	Increase from Single to Multi
Force at failure	Greatly increased	No	160%
Strain at failure	Increase	Yes	14%
Cyclic viscoplastic deformation	Decreased	Yes	−9.5%
Cyclic drift in sensor resistance	Greatly reduced	Yes	−54%
Monotonic region	Greatly increased	Yes	52%
Increased model accuracy for all strains	Greatly increased	Yes	300%

## Data Availability

Data available upon request.
